# Bioaccumulation, Ecotoxicity, and Microbial Responses in *Hoplobatrachus rugulosus* Tadpoles Following Co-Exposure to Imidacloprid and Microplastics

**DOI:** 10.3390/ani15131928

**Published:** 2025-06-30

**Authors:** Xinyu Hu, Sipu Zhu, Yiru Chen, Linxia Zhang, Huadong Tan, Chunyuan Wu, Xiaoying Zhang, Xiao Deng, Yi Li

**Affiliations:** 1 State Key Laboratory of Green Pesticide, Center for R&D of Fine Chemicals of Guizhou University, Guiyang 550025, China; huxinyu1209@163.com; 2 Environment and Plant Protection Institute, Chinese Academy of Tropical Agricultural Sciences, Haikou 571101, China; zhusipu11@163.com (S.Z.); cyrzh182@163.com (Y.C.); 13525527905@163.com (L.Z.); dx0928@foxmail.com (X.D.); wish.0310@163.com (Y.L.); 3 College of Resources & Environment, Huazhong Agricultural University, Wuhan 430070, China; 4 National Agricultural Experimental Station for Agricultural Environment, Danzhou 571737, China; 5 Haikou Experimental Station, Chinese Academy of Tropical Agricultural Sciences, Haikou 571101, China; zhangxiaoying_1990@163.com

**Keywords:** imidacloprid, microplastic, toxicokinetics, microbial diversity, tadpole, combined toxicity

## Abstract

**Highlights:**

- Q: How did IMI + MPs affect the tadpole LC_50_/TK parameters compared to their impact on growth? A: IMI + MPs negligibly affected the tadpole LC_50_/TK parameters compared to growth.
- Q: What was the difference in nuclear anomaly rates between tadpoles exposed to IMI alone and those exposed to IMI + MPs? A: IMI + MPs increased the nuclear anomaly rates in tadpoles compared to IMI.
- Q: How did the combination of IMI+ MPs influence the gut and skin microbiota in tadpoles? A: IMI + MPs induced a gut/skin microbiota diversity imbalance in tadpoles.
- Q: What was developed to assess the integrated effects of pollutants like IMI + MPs? A: A framework for a new multifaceted response evaluation was established to assess integrated effects.

**Simple Summary:**

This study investigated the individual and combined effects of imidacloprid and polyethylene microplastics on *Hoplobatrachus rugulosus* tadpoles, focusing on acute toxicity, growth, oxidative stress, and gut/skin microbiota. The results showed that while microplastics did not significantly alter the acute toxicity of imidacloprid, combined exposure markedly inhibited tadpole growth and induced oxidative stress and genotoxic damage. Furthermore, significant shifts in microbial community composition were observed, particularly in the gut and skin, with imbalances in dominant bacterial taxa. This study proposes a multi-tiered assessment framework to evaluate the combined toxicity of pollutants, providing new insights into their ecological risks. The findings highlight the need for the prudent use of pesticides and agricultural plastics to safeguard ecosystem health.

**Abstract:**

Agricultural organic pollutants have been identified as a key factor contributing to amphibian population decline, particularly during early developmental stages when tadpoles are frequently exposed to neonicotinoids (NEOs) and microplastics (MPs). In this study, *Hoplobatrachus rugulosus* tadpoles were exposed to imidacloprid (IMI: 0.045, 0.45, and 4.5 mg L^−1^) and polyethylene-derived MPs (10 mg L^−1^) from agricultural mulch films, both individually and in combination. We systematically evaluated acute toxicity, bioaccumulation, developmental and oxidative stress responses, and changes in the skin and gut microbiota. The results showed that the 96 h median lethal concentration (LC_50_) of IMI was 44.8 mg L^−1^ in the IMI-only group and was 40.5 mg L^−1^ in the IMI + MPs group, indicating the negligible impact of MPs on acute toxicity. However, in the highest co-exposure group (IMI4.5 + MPs), tadpole body length and weight decreased by 14.7% and 22.6%, respectively, alongside marked changes in oxidative stress, whereby catalase (CAT) and superoxide dismutase (SOD) activities were suppressed, while malondialdehyde (MDA) levels increased by 35%, indicating elevated lipid peroxidation. Furthermore, the micronucleus frequency in erythrocytes was significantly elevated, suggesting genotoxic effects. Microbial community analysis revealed significant shifts in the relative abundance of gut and skin microbiota under IMI + MPs exposure, with a notable enrichment of *Proteobacteria*, *Fusarium*, *Actinomycetota*, and *Bacteroidota*, indicating the disruption of host–microbiome interactions. This study proposes a comprehensive multi-tiered assessment framework encompassing environmental exposure, bioaccumulation, toxicological endpoints, oxidative stress biomarkers, and microbiome shifts. Our findings provide new mechanistic insights and quantitative evidence on the compound threats posed by IMI and MPs to amphibians in aquatic environments.

## 1. Introduction

Imidacloprid (IMI), which is a neonicotinoid insecticide (NEO), is widely detected in aquatic environments due to its high water solubility and persistence [1]. High concentrations of imidacloprid have been reported in agricultural areas (e.g., 294 μg L^−1^ in Australia [2] and 320 μg L^−1^ in the Netherlands [3]), usually occurring after rainfall. At the same time, research indicates that exposure to IMI affected oxidative stress and hormone levels in the tadpole brain [4]. Additionally, prolonged exposure to IMI led to a significant disruption in antioxidant enzymes and hormonal systems, which delayed metamorphosis in tadpoles [5]. High levels of these pollutants can significantly harm aquatic organisms [6], which might be the main cause underlying the sharp decline in the population and species diversity of amphibians and invertebrates globally [7].

Plastic mulch films are extensively used in agriculture for their ability to increase soil temperature, reduce moisture loss, and suppress weeds, thus enhancing crop yields [8]. However, these plastics degrade over time due to UV exposure, weathering, and physical abrasion, leading to the release of microplastics (MPs, <5 mm) into surrounding water bodies through rainfall and irrigation [9]. These MPs can accumulate in aquatic environments, with the highest reported global abundance in river water samples reaching 22,079 ± 134 particles L^−1^ [10]. These high levels of pollutants can significantly harm aquatic organisms [6]. Polyethylene MPs have been reported to cause histopathological changes [11], damage to tissues, and abnormalities in nuclear erythrocytes in tadpoles of Physalaemus cuvieri [12]. Given the widespread and concurrent use of neonicotinoid insecticides and plastic mulch films in modern agriculture, the co-occurrence of IMI and MPs in aquatic ecosystems is increasingly common. Agricultural runoff during rainfall events often transports both dissolved pesticides and degraded plastic particles into nearby water bodies, particularly in regions with intensive crop production. Empirical monitoring data have shown the simultaneous detection of MPs and IMI in surface waters near farmlands [13,14,15,16]. Moreover, polyethylene MPs originating from mulch films have demonstrated a strong sorption potential for pesticides like IMI, thereby increasing their environmental persistence and bioavailability [17]. This physicochemical interaction may facilitate the joint transfer of pollutants into aquatic organisms, increasing their combined toxicity.

While individual exposure to IMI or MPs presents significant risks, MPs usually appear in the aquatic environment as complex pollutants in combination with other environmental pollutants. Studies have increasingly highlighted the potential for synergistic effects when MPs are combined with other pollutants such as pesticides [18]. For instance, mixtures of MPs with organic pollutants like polycyclic aromatic hydrocarbons (PAHs) have shown synergistic toxicity in aquatic organisms [19]. Similarly, when MPs are co-exposed to pesticides, their combined toxicity may be significantly heightened, as has been observed in zebrafish (*Danio rerio*) exposed to both MPs and IMI [20]. Despite numerous studies on aquatic organisms, amphibians such as tadpoles, with unique physiological properties, remain understudied regarding combined exposure to MPs and IMI, leaving significant gaps in understanding how these contaminants interact at the physiological and ecological levels. As both aquatic and semi-terrestrial organisms with permeable skin, tadpoles are particularly vulnerable to environmental pollutants during development, affecting their physiology, metamorphosis, and survival rates. Their heightened sensitivity makes amphibians crucial bioindicators of ecosystem health [21]. Given the susceptibility of tadpoles to both IMI and MPs, it remains unclear how the combined exposure affects their physiological, developmental, and microbial responses compared to individual exposures. Moreover, to align regional reality with ecological sustainability goals, a multifaceted response evaluation framework is required to aid toxicological assessors and other relevant decision-makers in identifying the most sustainable risk threshold for the prevailing mixture in their high-application scenarios.

Here, we utilized the typical (sub)-tropical wild species of *Hoplobatrachus rugulosus* tadpoles as an experimental aquatic organism. They are widely distributed in southern China, Thailand, Vietnam, and other agricultural areas, with strong ecological representativeness [22]. Previous ecotoxicological studies have employed this species to assess parametric toxicity endpoints—including survival, growth, metamorphic timing, and biochemical stress—demonstrating their sensitivity to organic pollutants [23,24]. Additionally, their well-defined developmental stages and ease of laboratory culture support their use in controlled exposure studies. The objectives of this study were to (1) explore the acute toxicity (LC_50_) and bioconcentration (accumulation and elimination) of a typical NEO (IMI), MPs, and their mixture on *H. rugulosus* tadpoles; (2) determine the long-term growth development, antioxidant response, and the associated gut/epidermis microbial responses in *H. rugulosus* tadpoles exposed to IMI and MPs; (3) clarify the combined toxicity mechanisms of binary IMI and MPs. The findings of this study help establish the framework for a new multifaceted response evaluation for the rapid co-exposure assessment in high-application scenarios.

## 2. Materials and Methods

### 2.1. Chemicals and Reagents

The IMI standard (purity > 98.1%; CAS: 138261-41-3) was provided by J&K Scientific Co., Ltd. (Beijing, China). The physicochemical properties are shown in Supplementary Materials, Table S1. Imidacloprid-d_4_ (purity > 98%) was purchased from C.D.N Isotopes (Quebec, Canada) and used as a surrogate and internal standard (IS) for the analysis of IMI in target matrices. High-Performance Liquid Chromatography (HPLC)-grade acetonitrile, dichloromethane (DCM), and methanol were purchased from Thermo Fisher Scientific (Shanghai, China).

### 2.2. Sample Collection and the Preparation of MPs

Approximately one-year-aged polyethylene mulch (2 kg) was collected using cotton (plastic-free) gloves from a typical rice–vegetable rotation area in Chengmai County (19°44′20″ N, 110°00′27″ E), Hainan. After removing the plant debris and biological debris, the sample was placed in a cloth bag. The plastic film was first air-dried under natural conditions and then the particles attached to its surface were removed by shaking. Scissors were then used to cut the plastic film into pieces smaller than 1 × 1 cm. The entire plastic film was washed three times with a solution of nitric acid and hydrochloric acid for 5 min each time to remove the metal ions and organic particles adsorbed on the surface. Next, the plastic film was repeatedly washed with acetonitrile and n-hexane (*v*/*v* = 1:1) solution, and the adsorbed organic pollutants (such as polycyclic aromatic hydrocarbons, phthalates, and organochlorine pesticides) were removed with ultrasonic waves [19,25,26]. Once cleaned, the plastic film was broken with a grinder, and the fine plastic particles were filtered with an iron screen (0.63 mm) to obtain MPs of 500–1000 μm in size. The surface properties of MPs were characterized using scanning electron microscopy (Figure S1) and Fourier transform infrared spectroscopy (Figure S2).

### 2.3. Test Organism Acclimatization

Surface egg strings of *H. rugulosus* were purchased from an artificial breeding farm named Wusheng Frog Farm in Dazhipo Town, Meilan District, Haikou, Hainan Province, China (19°48′10″ N, 110°41′7″ E). According to a previously published study [27,28], egg masses were kept in clean water (25.0–33.5 °C, pH 6.50–7.50, and dissolved oxygen 5.0–7.0 mg L^−1^) until hatched. After hatching, tadpoles were fed daily with artificial food containing chicken egg yolk and dry vegetables purchased from Wusheng Frog Farm (Haikou, China). Tadpoles at stages 26–33 (GS26–33) with similar sizes (30 ± 1.5 mm) and weights (0.25 ± 0.05 g) were used for the experiments [29].

### 2.4. Acute Exposure Experiments

According to guideline 203 from the Organisation for Economic Co-operation and Development (OECD), as well as standard documentation (GB/T 31270.18-2014) in China [30,31], acute exposure experiments of IMI with or without MPs were conducted. A one-sixth concentration of MPs (10 mg L^−1^) was chosen in our study based on the recommended MP dose (60 mg L^−1^) for the co-exposure of amphibians to MPs with pesticides [32]. The MP concentration (10 mg L^−1^) used for the study was previously tested in amphibian tadpoles [33,34]. Then, 12 tadpoles were randomly selected and put in 1000 mL glass beakers containing 45, 75, 105, 135, 165, 195, and 225 mg L^−1^ IMI. Each treatment was conducted in triplicate. The tadpoles were not fed, and all exposure solutions were replaced every day. The dead tadpoles were immediately removed. The mortalities were recorded at 24, 48, 72, and 96 h to obtain the median lethal concentration (LC_50_).

### 2.5. Bioconcentration–Elimination of IMI in the Presence of MPs

#### 2.5.1. Experimental Design for Bioconcentration–Elimination Assays

In bio-uptake experiments, there were 8 treatment groups, as follows: (1) control (CK)—only water without IMI and MPs; (2) MP control (MPs)—10 mg L^−1^ MPs without IMI; (3) IMI0.045—low IMI (0.045 mg L^−1^) without MPs; (4) IMI0.45—medium IMI (0.45 mg L^−1^) without MPs; (5) IMI4.5—high IMI (4.5 mg L^−1^) without MPs; (6) IMI0.045 + MPs—low IMI (0.045 mg L^−1^) with MPs; (7) IMI0.045 + MPs—medium IMI (0.45 mg L^−1^) with MPs; and (8) IMI0.045 + MPs—high IMI (4.5 mg L^−1^) with MPs. Triplicates were conducted for all groups. Each group contained 30 tadpoles in a 1000 mL glass beaker with naturally filtered and aerated water. Bioconcentration–elimination experiments included multiple 4-day bioconcentration (0, 4, 10, 24, 48, 72, and 96 h) and 3-day elimination (100, 106, 120, 144, and 168 h) periods. During the exposure period, the solutions were renewed daily in a clean container to maintain a constant exposure concentration. In the elimination process, the tadpoles were transferred to clean filtered water without IMI or MPs. Culture water was renewed daily to avoid the tadpoles’ re-exposure to excreted MPs and IMI. The sample pretreatment procedures for IMI, as well as the LC-MS/MS conditions for IMI analysis, are provided in Text S1 in Supplementary Materials.

#### 2.5.2. Uptake and Depuration Kinetics

The uptake rate constant (*K*_u_), elimination rate constant (*K*_e_), and half-lives (*t*_1/2_) were obtained based on Equation (1), using a pseudo-first-order model [35].

(1) Ct=(KuKe)C0(1−e−ket)

where *K*_u_ is the uptake rate constant, *K*_e_ is the elimination rate constant, *C*_0_ is the IMI or MP concentration in tadpole tissue at the beginning of the experiment, and *C*_t_ is the IMI or MP concentration at time *t* (h). Parameter *K*_e_ is obtained according to Equation (2).

(2) $$C_{t}=C_{0}e^{-K_{e}t}$$

The *t*_1/2_ of IMI or MPs in tadpole tissues was calculated using Equation (3).

(3) $$t_{1/2}=\frac{\mathrm{In}2}{k_{e}}$$

The bioconcentration in tadpole tissue was assessed using the bioconcentration factor (BCF) of IMI or MPs according to Equation (4).

(4) $$\mathrm{BCF}=\frac{K_{u}}{K_{e}}$$

### 2.6. Exposure to Chronic Toxic Effects

#### 2.6.1. Experimental Design

The treatment groups were the same as the bio-uptake experiments (Section 2.5) and were used to conduct a 28-day chronic toxicity exposure experiment. Five tadpoles that survived at 7, 14, 21, and 28 days were collected for morphological, biochemical, and oxidative parameters, as well as associated intestinal/epidermis analyses.

#### 2.6.2. Morphological and Biochemical Parameters

For morphological endpoints, three tadpoles from each treatment were randomly fixed in 10% buffered formalin with phosphate-buffered solution (PBS). The weight was obtained by randomly weighing the tadpole for each treatment three times.

The activities of superoxide dismutase (SOD), catalase (CAT), malondialdehyde (MDA) (Comin Biotechnology Co, Ltd., Suzhou, China), and acetylcholinesterase (AChE) (Solarbio BC2025, China) were measured with commercial kits at the different exposure time points. Three random sub-samples with 3–5 tadpoles from each treatment were weighed (g) and homogenized (1:10, *w*/*v*) with a tissue grinder in ice-cold 25 mM sucrose and 20 mM Tris-HCl buffer (pH = 7.4) containing 1 mM EDTA to detect the enzymatic activities. Then, the samples were stored at −80 °C for further biomarker endpoint analysis. The results of all data were reported as the mean ± standard deviation (SD).

An integrated index—the IBRv2, established by Sanchez et al. [36]—was used to determine the toxicity stress. The growth and development (i.e., body length, body weight, micronucleus rate, and nuclear abnormality rate) and biomarker responses (SOD, CAT, MDA, and AChE) were all included in the IBRv2 in this study. The calculation for the IBRv2 index is given in Text S2.

#### 2.6.3. Intestinal and Epidermal Microbiome Analysis

Genomic DNA/RNA from the intestinal and epidermal microbiota of tadpoles at the final exposure period was extracted using the Cetyltrimethylammonium Bromide/Sodium Dodecyl Sulfate (CTAB/SDS) method [37]. After quality inspection, the V3-V4 regions of the bacterial 16S rDNA gene were amplified with primers 341F (CCTACGGGNGGCWGCAG) and 806R (GGACTACHVGGGTATCTAAT). Libraries were constructed using Ion Plus Fragment Library Kit 48 reactions (Thermo Fisher Scientific, Waltham, MA, USA) according to the standard protocols and were sequenced on an Ion S5TM XL platform. Valid data were filtered, and OTU (operational taxonomic unit) clustering was employed to analyze alpha (*α*) and beta (*β*) diversity, microbial community composition, and abundance. The functional predictions of OTUs and KEGG (Kyoto Encyclopedia of Genes and Genomes) pathways were performed using Tax4Fun analysis based on the 16S Silva database.

### 2.7. Quantitation Analysis of IMI and MPs in Surface Water and Tadpoles

To determine the uptake and elimination of IMI and MPs, the concentration of IMI and MPs in water was analyzed using a liquid chromatograph–mass spectrometer (UPLC-MS/MS) and the weighing/microscope-screening method [38,39,40], respectively. Tissue samples (1.00 ± 0.01 g) were extracted with a 5 mL acetonitrile solution in a 15 mL centrifuge tube. The extracted solution was purified using C_18_ (100 mg). The extract was rotary-evaporated to about 0.5 mL, and then the target compounds were measured and analyzed using UPLC-MS/MS after a 0.22 μm nylon membrane filtration. For the water sample, after filtering and centrifuging, the IMI in the water samples was determined using UPLC-MS/MS. The recoveries (90.56–102.03%) and precision (relative standard deviation < 5%) of IMI-spiked samples were used to assess the accuracy and precision of the analytical methods. The limits of detection (LODs) were 0.01–0.04 ng g^−1^ (biological tissue) and 0.002–0.05 μg L^−1^ (water) for the target compounds. For MPs in biological tissue, the counts were obtained using optical microscopy after digestion by hydrogen nitrate/H_2_O_2_ solution and membrane filtration (0.22 μm) based on the work of Di Fiore et al. [41]. Similarly to the count method of MPs in biological tissue, the amount of MP in the water was obtained after passing through a 0.22 μm membrane.

### 2.8. Statistics and Data Analyses

All statistical analyses were performed using IBM SPSS 22.0 (SPSS Inc., Chicago, IL, USA), and the data were presented as the mean ± SD. For morphological endpoints, samples were analyzed with a stereoscopic Arcano^®^ microscope and were photographed using a digital Moticam^®^ camera. The total length was measured using ImageJ software (https://imagej.net/ij/download.html (accessed on 15 June 2025)). The data distribution for normality and homogeneity of variances were verified using Kolmogorov–Smirnov and Levene tests. The Alpha diversity indexes (Chao and Shannon) were calculated using the software mothur (http://www.mothur.org/wiki/Calculators (accessed on 15 June 2025)). The interactive effects of IMI and MPs were identified using a two-way analysis of variance (ANOVA), with group differences being assessed using Tukey’s multiple tests. Redundancy analysis (RDA) was plotted using Canoco 5.0. Using the stepped-regression analysis method in SPSS 22.0, a quadratic polynomial model was established to describe the effects of the three selected variables, namely IMI, MPs, and the interactive items of IMI and MPs, on the oxidative stress, growth, nucleus damage, and gut/epidermal microbial diversity in tadpoles. Variables (*X*_1_, *X*_2_, and their interaction) were selected based on a significance threshold (*p* < 0.05 for entry, *p* > 0.10 for removal), with model fitness being validated via adjusted *R*^2^ (explaining >94% variance for all endpoints), F-test significance, and residual normality/homogeneity tests (Kolmogorov–Smirnov and Levene). The interaction term (*β*_ij_*X*ᵢ*X*_j_) quantified synergistic (positive *β*_ij_) or antagonistic (negative *β*_ij_) effects, cross-validated by two-way ANOVA and Tukey’s tests. Variance inflation factors (VIFs < 5) confirmed no multicollinearity, and the stepwise regression equations were reported with 95% confidence. The model is as follows:

*Y* = *β*_0_ + *β*_i_*X*_i_ + *β*_j_
*X*_j_ + *β*_ij_*X*_i_*X*_j_ + *ε* (5)

where *Y* represents the predicted value of the indices of tadpole oxidative stress, growth, nucleus damage, and gut/epidermal microbial diversity; *X* is the concentration of contaminants; *β*_0_ is the regression coefficient; *β*_i_ and *β*_j_ represent the strength of the effect of the different independent variables on the dependent variable; *β*_ij_ represents interaction effects (positive: synergistic; adverse: antagonistic; zero: additive); and *ε* represents the residual term. The stepwise regression equation has 95% confidence.

Moreover, the partial least squares structural equation model (PLS-SEM) conducted in Smart PLS (version 4.0) evaluated the interrelationship between body IMI content, gut MP content, and the gut and epidermis microbiota after IMI and MPs exposure. Differences were considered significant and very significant at *p* < 0.05 and *p* < 0.01, respectively.

## 3. Results and Discussion

### 3.1. Acute Toxicity Parameters (LC_50_) of IMI in the Presence of MPs

The estimated LC_50_ (Table 1) values for IMI were not significantly different (*p* > 0.05) in the presence or absence of MPs, indicating that the MPs (10 mg L^−1^), at common co-exposure levels in aquatic toxicity set to be higher than those in aquatic environments, did not alter the acute toxicity of IMI in tadpoles in terms of mortality. Notably, mortality in response to IMI with or without MPs was observed in tadpoles in a concentration-dependent manner (Table S2), indicating that as IMI concentrations increased, tadpoles had a shorter time to death and a higher mortality rate. These results showed that the presence of MPs did not significantly affect the acute toxicity of IMI to *H. rugulosus* tadpoles, including the LC_50_ and the concentration–effect relationship. This also indicates that the toxic effects of IMI are primarily determined by its concentration and not the MPs in the environment [42].

### 3.2. Uptake and Depuration Dynamics of IMI with or Without MPs

Regardless of the presence or absence of MPs, IMI rapidly reached equilibrium within approximately 10 h in the low-concentration exposure group (Figure S3A). During the 120 h clearance phase, the clearance of MPs showed a decrease of 78.6% in the low-concentration group, 80.8% in the medium-concentration group, and only 64.2% in the high-concentration group, indicating that MPs are not easily removed as compared with IMI. The results showed that the presence of MPs did not significantly increase the amount of IMI in *H. rugulosus* tadpoles at all concentrations (*p* > 0.05), as IMI is a hydrophilic compound (log *K*_OW_ = 0.6) and is unlikely to associate with MPs as a carrier, reducing its bioaccumulation in aquatic organisms [43]. Thus, MPs have a negligible effect on the bioaccumulation of IMI.

The relationship between tadpole MP levels and time was fitted to a two-compartment model using non-linear regression to estimate the uptake rate constants (*K*_u_) and elimination rate constants (*K*_e_). Under the exposure of MPs, the *K*_u_ of IMI0.045, IMI0.45, and IMI4.5 was estimated to be 0.0753 ± 0.0035 mL g^−1^ h^−1^ (mean ± SD), 0.0812 ± 0.0025, and 0.3667 ± 0.0195 mL g^−1^ h^−1^, respectively (Table 2); *K*_e_ values were estimated using non-linear regression to be 0.1287 ± 0.0200 mL g^−1^ h^−1^, 0.1711 ± 0.0113, and 0.4523 ± 0.0225 mL g^−1^ h^−1^, respectively (Table 2). Moreover, the bioaccumulation of IMI with MPs in tadpoles showed a better fit (*r*^2^ = 0.63–0.83; *p* < 0.001) than that in IMI without MPs (*r*^2^ = 0.48–0.51, *p* < 0.01). Among all *K*_u_ treatment values, high levels of IMI with MPs had the highest value at 0.3667 ± 0.0195 (mean ± SD) mL g^−1^ d^−1^ (Table 2). However, there was little difference among all *K*_e_ values in tadpoles (*p* > 0.05, Table 2). Overall, the *K*_u_ values were greater than the *K*_e_ values, resulting in higher BCFs for IMI in the presence of MPs. Additionally, given the different concentrations of IMI, the *K*_u_ values of the higher IMI level groups with MPs in tadpoles were higher than those with only IMI exposure (Table 2). In addition, it was found that the *K*_u_ of the high-concentration group was greater than that of the low- and medium-concentration groups, indicating that the rate of pollutant enrichment by *H. rugulosus* tadpoles increased as the concentration of IMI in the environment increased. As the IMI concentration increased, the *K*_e_ also increased, while the half-life (*t*_1/2_) decreased. This suggests that tadpoles are able to remove IMI from the body faster in high-pollutant concentration environments [44].

To further quantify the biokinetics of MPs in tadpoles, the temporal concentration of retained MPs in tadpole tissues was measured during both the uptake and elimination phases. The MPs-only group exhibited a rapid increase in body burden, reaching a peak concentration of 705 particles/L at 72 h, followed by a gradual decline during depuration (Figure S4). In the co-exposure groups (IMI0.045 + MPs, IMI0.45 + MPs, and IMI4.5 + MPs), MP accumulation was also observed, with lower peak concentrations compared to the MPs-only group, suggesting the possible modulation of MP retention by IMI. Despite partial elimination, all groups retained a substantial level of MPs at 168 h, confirming persistent internalization.

### 3.3. Chronic Toxicity Effects of IMI with and Without MPs on Tadpoles

#### 3.3.1. Growth/Development and Erythrocyte Nuclear Toxicity

**Figure 1:**
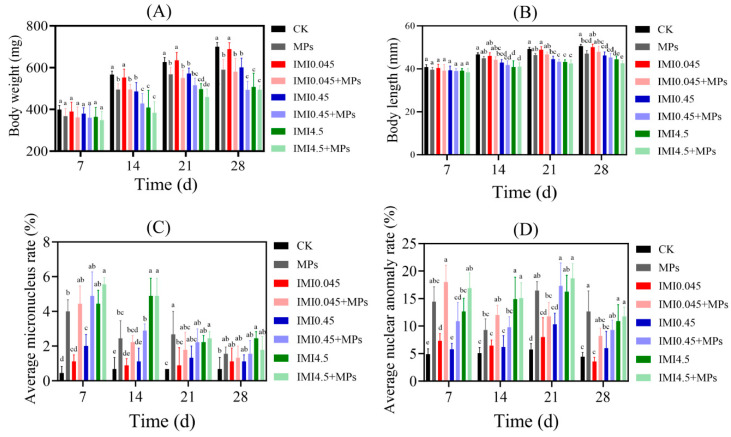
Morphological parameters or changes observed in *H. rugulosus* tadpoles exposed to IMI in the absence or presence of MPs after 28 days of exposure. Average micronucleus rate and average nuclear anomaly rate of IMI and MPs under single and combined exposure of *Hoplobatrachus rugulosus* tadpoles. (**A**) Body weight for IMI; (**B**) body length for IMI; (**C**) average micronucleus rate; (**D**) average nuclear anomaly rate. All weights are the fresh weight of tadpoles (*p* < 0.05, D and LSD test). Values represent the mean ± SD. Different lowercase letters above each bar denoted significant differences between treatments (*p* < 0.05; *n* = 6 animals/group).

Among the combined exposure groups, the IMI4.5 + MPs group showed a significant reduction of 6.1% in body length and 15.6% in body weight at day 7 (*p* < 0.05), as well as a reduction of 14.7% in body length and 22.6% in body weight by day 28 (Figure 1A,B). In addition, most of the tadpoles in the co-exposed groups had a lower body length and body weight than those in the IMI-exposed groups at the same concentration but were not significantly different from those in the MPs-exposed groups (*p* > 0.05, Figure 1A,B). The combined exposure of IMI and MPs significantly inhibited the length and weight of *H. rugulosus* tadpoles, with the IMI4.5 + MPs combined exposure group showing the strongest inhibitory effect. This indicates a synergistic effect of the two pollutants on the growth of tadpoles. The inhibition may be attributed to the accumulation of MPs in the intestinal tract following combined exposure, which creates a sense of satiety and reduces food intake, thereby suppressing growth and demonstrating a synergistic effect [45]. Thus, the higher the exposure concentration and the longer the exposure duration, the stronger the inhibition of tadpole length and weight, with MPs having a stronger inhibitory effect than IMI.

In the combined exposure groups, the micronucleus and nuclear anomaly rates were significantly higher than those in the control group and the IMI single-exposure groups at the same concentration (Figure 1C,D). On day 7, the IMI4.5 + MPs group showed the highest rates, with micronucleus and nuclear anomaly rates being 12.5 and 2.9 times higher than the control, respectively. Although these rates declined from day 14 onward, they remained significantly elevated by day 28 (*p* < 0.05), indicating that MP exposure further increased chromosomal damage and genotoxicity [46]. Moreover, the decline in these rates over time may be due to increased cytotoxicity causing cell death or the inhibition of normal cell division [47]. This suggests that MPs amplify the genetic damage caused by IMI, particularly in combined exposure scenarios.

#### 3.3.2. Oxidative Stress

**Figure 2:**
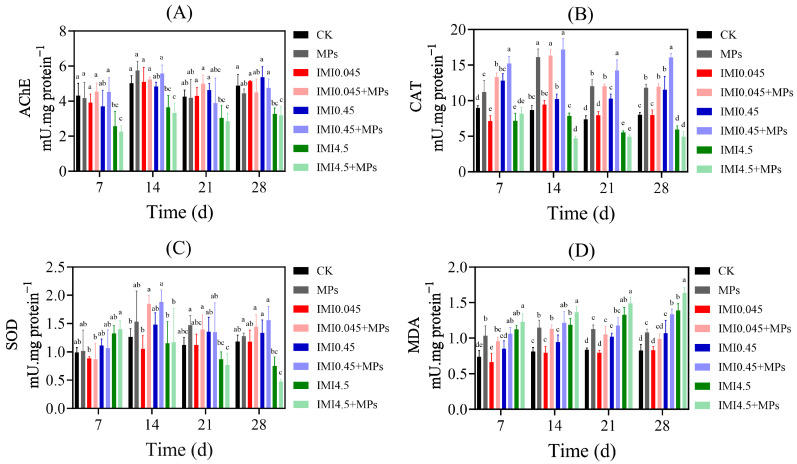
The influence of IMI on the biochemical parameters in tadpoles. (**A**) AChE activity. (**B**) CAT activity. (**C**) SOD activity. (**D**) MDA content. All weights are the fresh tadpole weight (*p* < 0.05, D and LSD tests). Values represent the mean ± SD. Different lowercase letters above each bar denoted significant differences between different treatments (*p* < 0.05).

**Figure 3:**
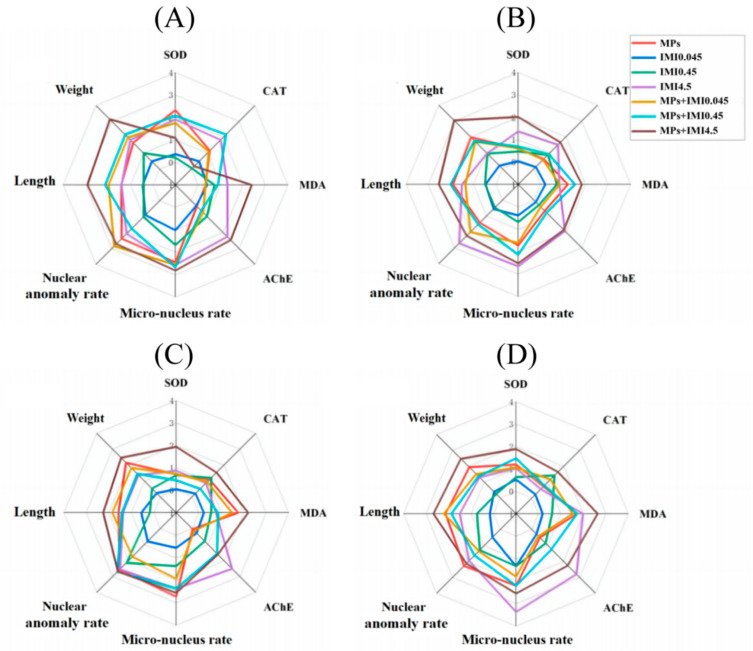
Results of integrated biomarker responses index (IBRv2) calculations based on growth/development, biochemical parameters, and erythrocyte nuclear abnormalities. Star plots for MPs, IMI0.045, IMI0.45, IMI4.5, IMI0.045 + MPs, IMI0.45 + MPs, and IMI4.5 + MPs on 7 (**A**), 14 (**B**), 21 (**C**), and 28 days (**D**).

In terms of AChE activity (Figure 2A), no significant changes were observed in tadpoles exposed to single MPs (*p* > 0.05). This may be due to the relatively large particle size of MPs, which primarily causes damage to organs such as the intestines and liver, thus having a less-pronounced impact on the nervous system [48]. However, compared to MPs, IMI exerts a stronger inhibitory effect on AChE activity. Under combined exposure, although AChE activity was generally lower than in the single MPs group, there was no significant difference compared to the IMI-exposed groups at the same concentration, indicating that IMI plays a dominant role in inhibiting AChE activity. Regarding antioxidant enzymes, both the single MPs group and most of the combined exposure groups showed a trend of initial induction, followed by the inhibition of CAT and SOD activities (Figure 2B and Figure 3C). From days 7 to 14, CAT and SOD activities increased, potentially reflecting an adaptive response by the organism to eliminate excess free radicals. However, as exposure continued, the amount of free radicals produced exceeded the organism’s capacity, leading to oxidative damage and a significant reduction in SOD and CAT activities [49,50]. In the IMI4.5 + MPs co-exposure group, SOD activity on days 14 and 21 was significantly lower than in the single-exposure groups, indicating that the presence of MPs exacerbated IMI-induced oxidative stress. These findings suggest a synergistic toxic effect of IMI and MPs [51]. The trend in MDA content changes was consistent with the alterations in antioxidant enzyme activities (Figure 2D). In both the single MPs group and most combined exposure groups, the MDA levels increased significantly in the later stages, with the IMI4.5 + MPs group showing the most pronounced elevation. This phenomenon can be attributed to the decline in antioxidant enzyme activity, resulting in excessive ROS accumulation and consequently higher levels of lipid peroxidation (MDA content) [52]. The synergistic interaction between IMI and MPs further intensified this oxidative stress, causing greater oxidative damage in tadpoles under combined exposure than with a single pollutant alone.

#### 3.3.3. Integrated Biological Response

Throughout the exposure period, the IMI4.5 and IMI4.5 + MPs exposure groups showed strong induction or inhibition effects on growth and development, antioxidant systems, the nervous system, and genotoxicity. Aside from the IMI0.045 exposure group, which had a relatively minor impact on the eight biomarkers, the effects on IBR were generally similar among the other five exposure groups (Figure 3). Notably, the IBR value of the IMI4.5 + MPs exposure group increased significantly and was markedly different from that of the IMI0.045 + MPs exposure group (*p* < 0.01). Meanwhile, the higher the IBR indices of IMI and MPs, the greater their combined toxicity to *H. rugulosus* tadpoles. In the combined exposure groups, there was a dose-dependent relationship between IBR values and pollutant concentrations, with higher concentrations causing greater harm to the tadpoles [53]. Furthermore, the IBR value of the IMI4.5 + MPs exposure group was higher than that of the IMI4.5 exposure group and the MPs exposure group, indicating a synergistic effect of the two pollutants in the IMI4.5 + MPs combination, which led to more severe toxicity in the tadpoles.

### 3.4. Associated Microbial Diversity Responses

#### 3.4.1. Gut Microbial Diversity

**Figure 4:**
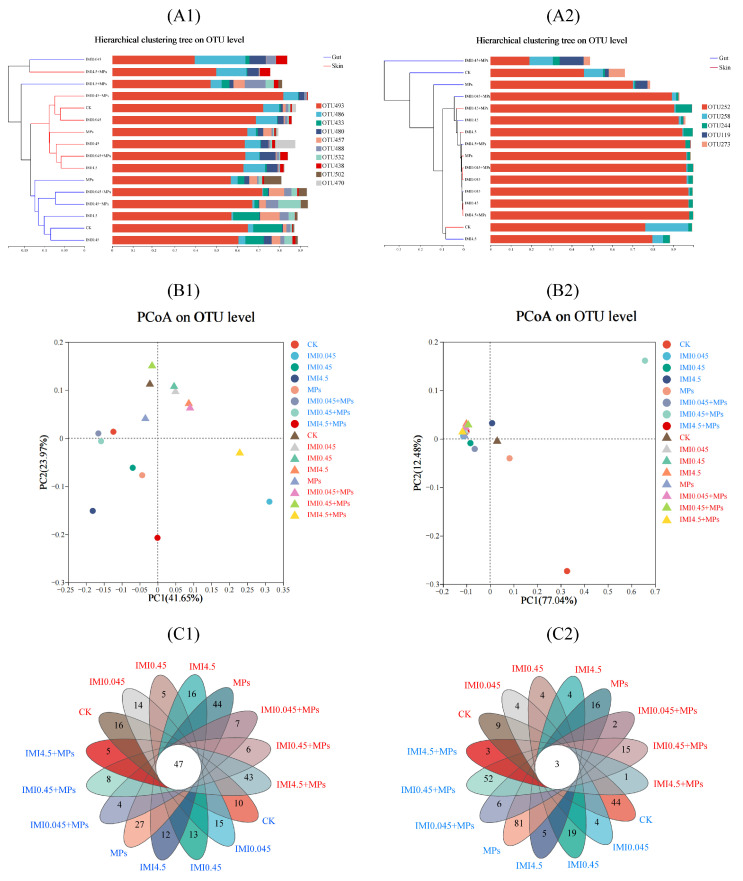
Differences or similarities in the microbial community composition between different IMI treatment groups ((**A1**–**C1**) represents bacteria; (**A2**–**C2**) represents fungi) in the absence and presence of MPs after 28 days of exposure. (**A**) Hierarchical clustering of Bray–Curtis distance generated according to the operational taxonomic unit (OTU) of the tadpole microbiome. (**B**) Principal coordinate analysis (PCoA) indicating differences in the gut microbiome between CK and NEO or individual MP groups. (**C**) The Venn diagrams indicate the number of unique bacterial OTUs in the tadpole gut microbiota among individual and combined treatments. The blue font represents the gut, while the red font represents the epidermis.

Significant differences in the bacterial communities in the IMI control and IMI + MPs groups clustered separately, indicating that MPs changed the bacterial diversity pattern. No clear differences (*p* > 0.05) were observed for IMI regardless of the absence or presence of MPs. These differences between the control with only IMI/MPs, low NEO concentrations with MPs, and high IMI concentrations with MPs were also highly consistent with the results in a cluster dendrogram (Figure 4A). These results show that IMI exposure (regardless of IMI concentration) combined with MPs induce a significant influence on the diversity of the gut microbiota in tadpoles [54].

The differences in the intestinal microbial composition based on QIIME2 (2023.6) were explored to characterize the tadpole gut community structure, as shown in Figure 4. In general, the significant differences in the gut microbial community structure occurred in the high IMI with MPs groups. At the phylum level, these differences occurred mainly through variations in the relative abundances of keystone taxa, e.g., *Proteobacteria*, *Bacteroidetes*, and *Tenericutes*, which differed from the control group, where the proportions of *Proteobacteria* (78.8%) and *Fusobacteria* (18.4%) were dominant in the 4.5IMI + MPs-treated group (Figure 4). Also, the relative abundances of *Proteobacteria* and *Bacteroidetes* were increased in the other IMI with MPs exposure groups. Similar results have been observed in other reported studies [55]. For example, the relative abundance of keystone taxa (such as *Proteobacteria*, *Firmicutes*, and *Bacteroidetes*) in *Corbicula fluminea* was increased by co-exposure (MPs; 10 mg L^−1^ with glyphosate for 28 days). Alterations in the keystone taxa were associated with the overall health of the intestinal tract, as well as the growth and development of the tadpole, and have been frequently observed in aquatic organisms exposed to MPs or insecticides [56,57].

Regardless of IMI, the IMI0.45 combined with MPs treatment presented more specific OTUs than other treatments (Figure 4C). These data suggested that the medium IMI with MPs group could reduce the number of OTUs in the intestinal and epidermal microbiota, whereas IMI0.045 with MPs increased the number of OTUs in the intestinal microbiota. For the IMI-only treatments, the Chao index (Figure S5a) and the Shannon index (Figure S5b) in the IMI4.5 group were lower than those of the IMI0.045/IMI0.45 exposure groups; however, there were no significant differences among these treatments (*p* > 0.05). For the IMI with MPs treatment groups, the Chao index and Shannon index in the IMI0.45 with MPs groups were the lowest compared with the other IMI with MPs treatment groups (*p* < 0.01). Therefore, the IMI combined with MPs groups seemed to lead to a significant decrease in the Chao index and Shannon index compared with the IMI without MPs groups.

#### 3.4.2. Epidermis Microbial Diversity

At the phylum level, *Proteobacteria*, *Bacteroidetes*, and *Actinobacteria* dominated the tadpole epidermis, accounting for 51.03%, 18.99%, and 21.78% of the total sequencing, respectively (Figure 4). *Proteobacteria* abundance increased significantly after all exposure treatments (*p* < 0.05). IMI with MP exposure reduced levels of *Bacteroidetes* and increased levels of *Actinobacteria*, showing significant differences compared to the control group (*p* < 0.05). LDA (linear discriminant analysis) and LEfSe (LDA effect size) analyses revealed distinct microbial biomarkers, including *Flavobacteriaceae* and unidentified_Chloroplast in the control group, *Demequinaceae* in the IMI groups, and *Rhodobacteraceae* and *Microbacteriaceae* in the IMI with MPs groups.

At the genus level, significant differences were observed among *Microbacterium*, *Pseudoalteromonas*, *Demequina*, *Vibrio*, and *Tenacibaculum* (Figure 4). The high abundance of Lactobacillus, unidentified_Chloroplast, and Acidothermus in the control group was significantly reduced in the IMI and IMI with MPs groups (*p* < 0.05). Compared to the IMI group, the proportions of *Vibrio*, *Tenacibaculum*, *Microbacterium*, *Bradyrhizobium*, and *Demequina* increased in the IMI with MPs groups. The abundance of *Vibrio* and *Tenacibaculum* was significantly higher in the IMI groups than in the control group (*p* < 0.05), while *Microbacterium* and *Demequina* were significantly higher in the high IMI with MPs groups than in both control and IMI groups (*p* < 0.05). PCoA analysis based on unweighted UniFrac distances showed clear separations in the intestinal and epidermal microbiotas among the control, IMI, and IMI with MPs groups (Figure 4B). These results suggest that IMI and MPs exposure significantly alters microbial community structures in tadpoles, reducing beneficial genera and increasing stress-related taxa, indicating the strong influence of these environmental stressors on microbial ecology [58].

The significant differences in gut and epidermal microbial responses to environmental exposures highlight their varying sensitivities to complex pollution, which may be closely related to the exposure pathways and health status of tadpoles. The gut microbiome appears to show a greater sensitivity due to the direct ingestion of pollutants, with more rapid changes in environmental stress [59], especially as it serves as a primary sink for MPs. However, the epidermal microbiome also represents an important factor due to its direct contact with IMI and MPs. Therefore, studying the stress-induced changes in microbial diversity in both gut and epidermal microbiomes is crucial for assessing the health and environmental adaptability of amphibians [60]. Furthermore, the structure of the epidermal microbiome changes with the developmental stages of amphibians and their environmental interactions. Consideration of the epidermis with gut microbiomes can complement the responses observed only in the gut, which can comprehensively reflect sensitivities to various ecological stresses [61].

### 3.5. Relationships Among Toxicity Response Indicators and Their Complex Toxicity Interaction Mode

The RDA results showed that the total variance was 96.0, explaining 76.6% of the variance, with the variables of tadpole enzyme activity, growth, development, and erythrocyte nucleotoxicity contributing more to the explained variance of MPs, time, and IMI (Figure S6a and Table S3). The first two axes explained 55.99% of the overall variance (Figure S6a). The enzyme variables accounted for 22.9% of the explained variance. The most relevant variables to axis 1 were CAT and AChE. AChE was positively correlated with IMI, but negatively correlated with MPs, as previously described; additionally, IMI increased AChE activity, suggesting nervous system stress. CAT was negatively correlated with MPs and IMI, but positively correlated with time, indicating that increased CAT activity over time indicated tadpole adaptation to pollutants. MPs and IMI together inhibited growth rates and increased erythrocyte nuclear abnormalities, indicating genotoxicity. MPs alone inhibited antioxidant enzyme activity and damaged erythrocyte nuclear structure, highlighting oxidative stress and genotoxic effects [62].

SEM assessed the interrelationships between intestinal MPs concentration, IMI concentration in tadpoles, and intestinal and epidermal microbial communities. The model explained 62.6% of intestinal MPs concentration, 94.4% of IMI concentration in tadpoles, 58.9% of the intestinal microbial community, and 27.3% of the epidermal microbial community (Figure S6b). Epidermal microbes were affected differently by single and composite treatments (Figure S6b). Treatment negatively impacted gut microbial diversity (*p* < 0.05), indicating interference with microbial balance, likely due to contaminants. The intestinal MPs concentration positively affected IMI accumulation in tadpoles (*p* < 0.01), which is consistent with the above results that suggest that MPs influence IMI metabolism and absorption [63]. Changes in gut microbial community positively affected the epidermal community (*p* < 0.05), implying microbial interactions or mutual regulation. A positive correlation between intestinal MPs and gut microbial community (*p* < 0.01) suggests that MPs affect gut microbes directly or indirectly, possibly by providing new habitats or altering intestinal conditions.

The stepwise regression analyses results showed that IMI and MPs, as well as their interactions, significantly affected several biological characteristics of tadpoles (Table 3). Multifactor regression analysis indicated that IMI (*X*_1_) had a negative effect on CAT and a positive effect on MDA, SOD, and AChE, suggesting that IMI inhibits CAT activity but promotes MDA content, as well as SOD and AChE activity. MPs (*X*_2_) negatively affected SOD activity. Interaction terms (*X*_1_*X*_2_) were negative for the CAT- and AChE-related indices, indicating that combined exposure to IMI and MPs decreased antioxidant enzyme activities, increasing damage to tadpoles [64]. Meanwhile, the regression equations for growth and development indicators showed negative values for both *X*_1_ and *X*_2_, indicating that increased IMI and MPs decreased tadpole body length and weight, which is consistent with the results of our above study, likely due to impaired digestion or reduced food intake. The interaction terms (*X*_1_*X*_2_) of IMI and MPs were positive for growth indicators, suggesting combined IMI and MP exposure promoted growth. However, interaction terms were negative for gut bacterial Shannon and Simpson indices, as well as the intestinal fungal Shannon index, suggesting that combined exposure to IMI and MPs negatively affected microbial diversity.

### 3.6. Multifaceted Response Evaluation Framework

**Figure 5:**
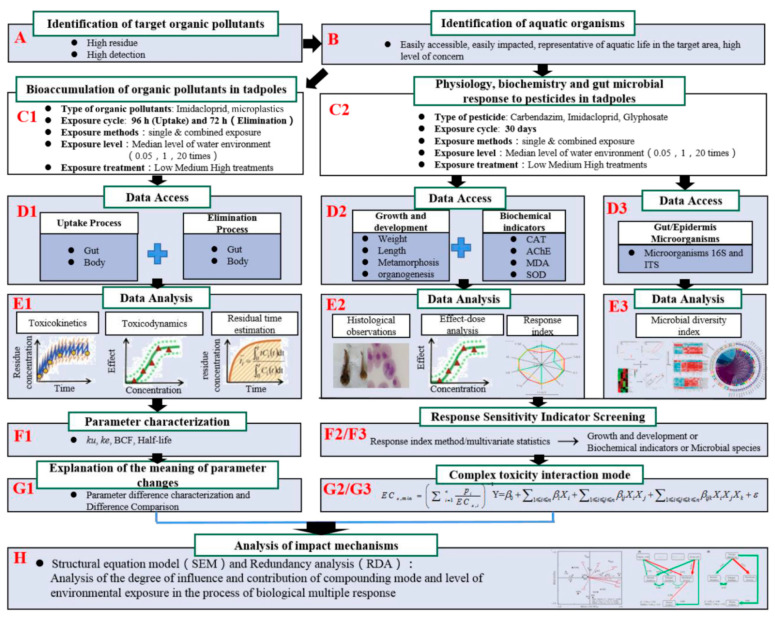
Flow diagram of simplified multifaceted evaluation framework for the combined toxicity of an NEO and MPs to aquatic tadpoles. CAT: catalase; AChE: acetylcholinesterase; MDA: malondialdehyde; SOD: superoxide dismutase; *k*_u_: uptake rate constant; *k*_e_: elimination rate constant (*K*_e_); BCF: bioconcentration factor. (A) Identification of target organic pollutants; (B) Identification of aquatic organisms; (C1) Bioaccumulation of organic pollutants in tadpoles; (C2) Physiology, biochemistry and gut microbial response to pesticides in tadpoles; (D1) Data access: uptake process, elimination process; (D2) Data access: Growth and development, biochemical indicators; (D3) Data access: Gut/epidermis and microorganisms; (E1) Data analysis: Toxicokinetics, toxicodynamics and residual time estimation; (E2) Data analysis: Histological observations, effect-dose analysis and response index; (E3) Data analysis: Microbial diversity index; (F1) Parameter characterization; (F2) (F3) Response sensitivity indicator screening; (G1) Explanation of the meaning of parameter changes; (G2) (G3) Complex toxicity interaction mode; (H) Analysis of impact mechanisms.

The framework starts with environmental exposure and covers bioconcentration processes, toxicity characterization, and oxidative stress responses in tadpoles (Figure 5). In addition, we explored the response of the gut and epidermal microbial communities to chemical contamination. The epidermis acts as a critical interface between tadpoles and their environment, facilitating physical protection and chemical exchanges. Thus, the epidermis is incorporated into the assessment framework due to its role in chemical exchange and physical protection.

In this assessment framework, we used a new multi-parametric approach to understand the effects of co-exposure to IMI and MPs on tadpoles by integrating behavior, physiology, molecular biology, and microbiology. The framework is not limited to traditional biological metrics, such as mortality and growth inhibition. An analysis of bioconcentration processes demonstrates the dynamics of IMI and MP accumulation in tadpoles and reveals the possible long-term effects of this accumulation on tadpole health. In addition, our use of oxidative stress biomarkers, such as antioxidant enzyme activity assays, provided sensitive indicators for assessing cellular damage induced by chemical contaminants. Through in-depth studies of gut and epidermal microbial communities, we revealed changes in microbial diversity and community structure. Changes in the microbiome may not only affect nutrient uptake in tadpoles but can also alter their sensitivity to environmental pollutants.

The developed assessment framework underscores that the selection of exposure species is based on the residual characteristics of real-world scenarios. This approach highlights the pronounced effects of both single and compound exposures on aquatic organisms and microbial communities, especially in environments characterized by high detection rates and compounded contamination. Our choice of species aligns with the target substances that are focused on in certain toxicological studies, such as glyphosate and polyethylene microplastics (PE MPs) [65,66]. These substances exhibit high detection and residue levels in the actual environment and demonstrate toxic effects under experimental conditions when combined. The combined effects of MPs and organic pollutants can pose several ecological effects, including toxicity, bioaccumulation and biomagnification, physical effects, the alteration of microbial communities, and ecosystem disruption [67]. Therefore, by intentionally adjusting or modularly increasing/decreasing specific parameters, test methods, and assessment contents in the assessment framework, the assessment can be customized for different environmental media and pollutant types.

## 4. Conclusions

This study comprehensively evaluated the multifaceted toxic effects of IMI and aged MPs on *H. rugulosus* tadpoles, encompassing acute toxicity, bioaccumulation, developmental and oxidative stress responses, as well as gut and epidermal microbiota alterations. Although MPs did not significantly enhance IMI bioaccumulation, both pollutants—individually and jointly—inhibited tadpole growth, with MPs exerting a more potent suppressive effect. IMI demonstrated neurotoxicity by significantly inhibiting AChE activity and disrupting antioxidant enzymes, whereas MPs primarily intensified oxidative stress. Furthermore, combined exposure elevated genetic damage and induced notable dysbiosis in microbial communities, indicating complex pollutant interactions.

Future studies should further explore the mechanisms of interactions between different types and concentrations of pollutants. Toxicological results based on actual observations should be used as evidence to refine the study, while new histological techniques can be introduced as modular or complementary tools for high-throughput analyses to provide new insights from multiple dimensions. However, it is important to note that the current study did not monitor several critical developmental milestones, such as limb formation, metamorphic progression, and specific developmental timelines. This limitation may reduce the ability to fully elucidate how co-exposure disrupts amphibian ontogeny. Therefore, future research should incorporate more comprehensive developmental tracking throughout the full life cycle of amphibians. Considering the prevalent mixed state of pollutants in the environment, future research should also focus more on the ecological risk assessment of combined multi-pollutant (*n* ≥ 3) exposure in order to more accuratgely predict and manage the potential impacts on biodiversity and ecosystem health.

## Figures and Tables

**Table 1:** The LC_50_ values of an NEO with or without MPs in *H. rugulosus* tadpoles at 24, 48, 72, and 96 h.

Test Chemicals	MPs (10 mg L^−1^)	Media Lethal Concentration (LC_50_) Values (mg L^−1^) with 95% Confidence Intervals							
		24 h LC_50_	*R* ^2^	48 h LC_50_	*R* ^2^	72 h LC_50_	*R* ^2^	96 h LC_50_	*R* ^2^
IMI	−	143.9	0.9316	113.6	0.9115	71.8	0.8762	44.8	0.8281
IMI	+	112.1	0.9737	99.9	0.9749	55.3	0.9922	40.5	0.9791

*R*^2^: determination coefficient of fitting model for LC_50_ estimation; IMI: imidacloprid.

**Table 2:** Kinetic parameters of the uptake and depuration of IMI in the absence and presence of MPs in *H. rugulosus* tadpoles. For IMI, the values of *k*_u_ and *k*_e_ in the body, as well as MPs in the body, were estimated simultaneously using non-linear regression.

IMI Concentration (mg L^−1^)	MPs (mg L^−1^)		IMI in Body			
		Treatments	*K*_u_ (h^−1^)	*K*_e_ (h^−1^)	BCF	*t*_1/2_ (h)
0	0	Control	–	–	–	–
0	10	–	–	–	–	–
0.045	0	IMI0.045	0.0909 ± 0.0033	0.1504 ± 0.0309	0.6045	4.61
0.045	10	IMI0.045 + MPs	0.0753 ± 0.0035	0.1287 ± 0.0200	0.5852	5.39
0.45	0	IMI0.45	0.0893 ± 0.0018	0.1917 ± 0.0169	0.4658	3.62
0.45	10	IMI0.45 + MPs	0.0812 ± 0.0025	0.1711 ± 0.0113	0.4745	4.05
4.5	0	IMI4.5	0.3391 ± 0.0138	0.4260 ± 0.0281	0.7960	1.63
4.5	10	IMI4.5 + MPs	0.3667 ± 0.0195	0.4523 ± 0.0225	0.8108	1.53

*K*_u_: conditional uptake rate constant; *K*_e_: conditional elimination rate constant; BCF: bioconcentration factor; t_1/2_: half-time elimination; IMI: imidacloprid; MPs: microplastics.

**Table 3:** Relationships between the body ROS content, AChE content, body weight/length inhibition rate, nucleus damage, gut/epidermal microbial diversity of tadpoles (*Y*), and the exposure of IMI (*X*_1_) and MPs (*X*_2_).

Response Indices	Multiple Regression Model	Adjusted *R*^2^
SOD	*Y* = 0.73982 + 0.01605*X*_1_ − 0.00289*X*_2_ + 0.00126*X*_1_*X*_2_	0.99999
CAT	*Y* = 9.18858 − 0.65982*X*_1_ + 0.59738*X*_2_ − 0.18605*X*_1_*X*_2_	0.95911
MDA	*Y* = 0.88662 + 0.11455*X*_1_ + 0.02216*X*_2_ + 0.00152*X*_1_*X*_2_	0.99947
AChE	*Y* = 3.10489 + 0.26058*X*_1_ + 0.10801*X*_2_ − 0.05683*X*_1_*X*_2_	0.93878
Body length	*Y* = 49.69478 − 1.46457*X*_1_ − 0.25774*X*_2_ + 0.03647*X*_1_*X*_2_	0.97144
Body weight	*Y* = 681.71573 − 43.73941*X*_1_ − 9.79472*X*_2_ + 0.68524*X*_1_*X*_2_	0.99962
Micronucleus rate	*Y* = 0.11969 + 0.0732*X*_1_ + 0.03262*X*_2_ − 0.00921*X*_1_*X*_2_	0.99994
Nuclear abnormality rate	*Y* = 0.57716 + 0.1524*X*_1_ + 0.08844*X*_2_ − 0.01835*X*_1_*X*_2_	0.99789
Gut bacterial Shannon index	*Y* = 0.82536 + 0.03047*X*_1_ + 0.006*X*_2_ − 0.00148*X*_1_*X*_2_	0.99949
Gut bacterial Simpson index	*Y* = 0.34895 + 0.00134*X*_1_ + 0.00989*X*_2_ − 0.00458*X*_1_*X*_2_	0.99965
Gut fungal Shannon index	*Y* = 1.08521 − 0.04488*X*_1_ + 0.10239*X*_2_ − 0.04302*X*_1_*X*_2_	0.94752
Gut fungal Simpson index	*Y* = 0.67644 − 0.00287*X*_1_ − 0.02236*X*_2_ + 0.01003*X*_1_*X*_2_	0.99537
Epidermis bacterial Shannon index	*Y* = 1.5094 + 0.06135*X*_1_ + 0.00408*X*_2_ + 0.00800*X*_1_*X*_2_	0.99523
Epidermis bacterial Simpson index	*Y* = 0.48299 − 0.01723*X*_1_ + 0.00251*X*_2_ − 0.00304*X*_1_*X*_2_	0.99957
Epidermis fungal Shannon index	*Y* = 0.3714 − 0.05632*X*_1_ − 0.0099*X*_2_ + 0.00217*X*_1_*X*_2_	0.99874
Epidermis fungal Simpson index	*Y* = 0.82536 + 0.03047*X*_1_ + 0.006*X*_2_ − 0.00148*X*_1_*X*_2_	0.99949

*R*^2^: coefficient of determination; SOD: superoxide dismutase; CAT: catalase; MDA: malondialdehyde; AChE: acetylcholinesterase.

## Data Availability

The data presented in this study are available upon reasonable request from the corresponding author.

## Supplementary Materials

The following supporting information can be downloaded at https://www.mdpi.com/article/10.3390/ani15131928/s1. Figure S1: Scanning electron microscopy (SEM) micrographs showing not aged (a1–a3) polyethylene MPs, aging MPs (b1–b3) at different magnification; Figure S2: Fourier transform infrared spectroscopy shows aging polyethylene MPs (A-MPs), MPs, CK, IMI; Figure S3: Residual IMI concentrations in tadpoles. ((A) low concentration group; (B) medium concentration group; (C) high concentration group); Figure S4: Residual MPs concentrations in tadpoles; Figure S5: The α diversity in the different exposure groups, including Chao (a) and Shannon indices (b); (a1,b1) represent bacteria, (a2,b2) represent fungi; Figure S6: (a) Redundancy analysis (RDA) results for IMI and MPs co-exposure treatment and their toxicity response variables. (b) Numerical values near lines in the structural equation model (SEM) path analysis are standardized path coefficients. Pathway significance: * *p* < 0.05, ** *p* < 0.01, which are indicated by bold font; Table S1: Physicochemical properties of imidacloprid (IMI); Table S2: Mortality of *H. rugulosus* when exposed to the different concentration of IMI in presence or absence of MPs over 24, 48, 72, 96 h; Table S3: Analysis results of the redundancy analysis (RDA) grouped by oxidative stress markers; Text S1: Pretreatment and instrument analysis of NEOs; Text S2: The integrated biomarker responses (IBRv2).
