# Supplementary material for: Bioaccumulation, Ecotoxicity, and Microbial Responses in Hoplobatrachus rugulosus Tadpoles Following Co-Exposure to Imidacloprid and Microplastics
Source: Animals (Basel). 2025 Jun 30;15(13):1928. doi: 10.3390/ani15131928 (PMC12249034; doi:10.3390/ani15131928)
Supplement: Supplementary file 1 [file animals-15-01928-s001.zip › animals-3677119-supplementary.pdf]

Supplementary Materials

Bioaccumulation, Ecotoxicity, and Microbial Responses in *Hoplobatrachus rugulosus* Tadpoles Following Co-Exposure to Imidacloprid and Microplastics

Xinyu Hu [1,2], Sipu Zhu [2,3], Yiru Chen [2,3], Linxia Zhang [2,3], Huadong Tan [2,4,*],Chunyuan Wu [2,4,*], Xiaoying Zhang [5], Xiao Deng [2,4] and Yi Li [2,4]

[1] State Key Laboratory of Green Pesticide, Center for R&D of Fine Chemicals of Guizhou University, Guiyang 550025, China

[2] Environment and Plant Protection Institute, Chinese Academy of Tropical Agricultural Sciences, Haikou 571101, China

[3] College of Resources&Environment of Huazhong Agricultural University, Wuhan 430070, China

[4] National Agricultural Experimental Station for Agricultural Environment, Danzhou 571737, China

[5] Haikou Experimental Station, Chinese Academy of Tropical Agricultural Sciences, Haikou 571101, China

[*] Corresponding author:

Huadong Tan, Environment and Plant Protection Institute, Chinese Academy of Tropical Agricultural Sciences, Haikou, China; E-mail: tanhuadong1991@163.com.

Chunyuan Wu, Environment and Plant Protection Institute, Chinese Academy of Tropical Agricultural Sciences, Haikou, China; E-mail: wuchunyuangz@126.com.

Zip/Postal code: 571101; TEL: +86 898 66969272.

Text S1: Pretreatment and instrument analysis of NEOs

After thawing the tadpole samples at 25±1°C, they were placed in a 15-ml polypropylene centrifuge tube, and 10 ml of acetonitrile was added. The mixture was homogenized with an electric tissue homogenizer for 1 min, followed by the addition of 2 g of anhydrous magnesium sulfate and 0.5 g of sodium chloride. The mixture was shaken for 1 min, then subjected to ultrasonic extraction for 3 min, and centrifuged at 3000 rpm for 5 min. The acetonitrile layer of the supernatant was transferred to a sample purification column containing 0.2 g of C_{18} and 0.4 g of anhydrous magnesium sulfate, vortexed for 1 min, and then centrifuged at 3000 rpm for 10 min. The purified extract was then rotary-evaporated to dryness at 40°C, reconstituted in acetonitrile to 1 mL, and filtered through a 0.22-μm filter membrane. The extract was transferred to a 1.5-mL brown bottle, and 10 uL of internal standard was added for UPLC-MS/MS analysis. IMI is highly water-soluble. For the analysis of IMI content in aqueous solutions, water samples were directly filtered through a 0.22-μm filter membrane, and 10 μL of internal standard was added for UPLC-MS/MS analysis.

The chromatographic column used was an ACQUITY UPLC HSS T3 (100 mm x 2.1 mm x 1.8 μm), with a column temperature of 40°C and an autosampler temperature of 25°C. The mobile phase consisted of methanol (Phase A) and a 0.1% formic acid aqueous solution (Phase B), with an injection volume of 5 μL and a run time of 6.0 min. Analysis was performed in electrospray ionization positive mode (ESI+), with a voltage of 5500 V and a temperature of 500°C. Multiple Reaction Monitoring (MRM) mode was used; capillary voltage was 3.2 kV; nebulizer pressure was 310 kPa; gas for nitrogen-solvent wass at 250°C, 800 L/h; cone voltage was 15 V;

and cone gas (nitrogen) flow was 5 L/h.

Text S2: The integrated biomarker responses (IBRv2)

Growth parameters (body length and weight) were incorporated into the IBRv2 calculation alongside biochemical biomarkers to provide a multi-tiered toxicity assessment. Growth inhibition reflects long-term organismal-level impairment, while oxidative and neurotoxic biomarkers capture short-term sublethal cellular effects.

The standardized value of each biomarker was obtained according to Eq. (1).

$$Y_i = \log(\frac{X_i}{X_0}) \quad (1)$$

where X_i is the average value of each biomarker and X_0 is the average value for the biomarker in the control treatment. Y_i was then standardized [Eq. (2)] by using μ and σ, which are the average value and the standard deviation of Y_i.

$$Z_i = \frac{(Y_i - \mu)}{\sigma_0} \quad (2)$$

A deviation index (A) is obtained by subtracting the average value of the control group (Z_0) to the average value of the treated groups (Z_i) [Eq (3)].

$$A_i = Z_i - Z_0 \quad (3)$$

Finally, the IBR values were achieved according to the following equation:

$$IBR = \sum(|A|/n) \quad (4)$$

where n is number of biomarkers. The star plots were applied to represent the scores obtained at each exposure time. The IBR values and star plots were conducted in Excel software (Microsoft, Washington DC, USA).

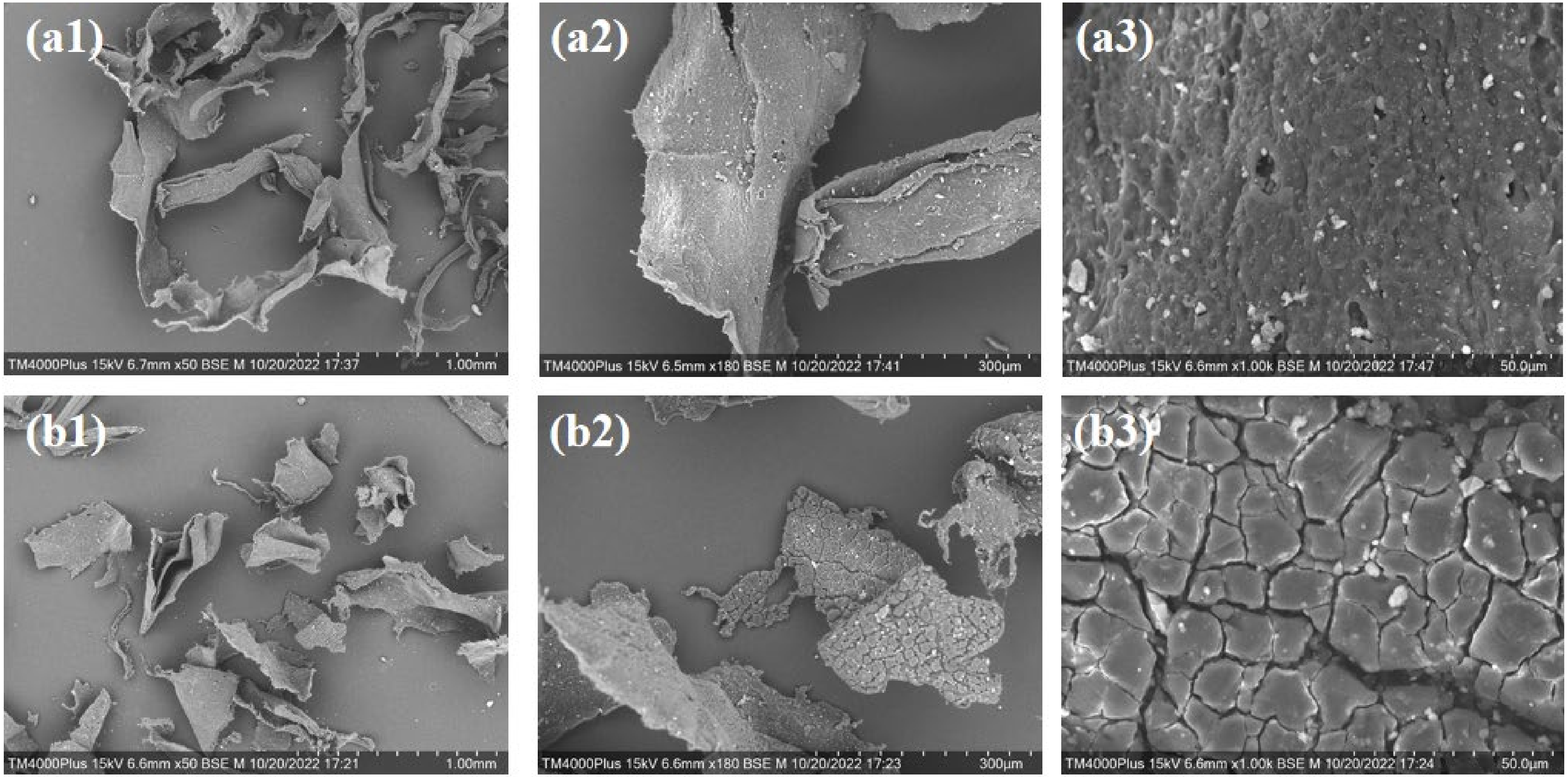

Figure S1. Scanning electron microscopy (SEM) micrographs showing not aged (a1–a3) polyethylene MPs, aging MPs (b1–b3) at different magnification.

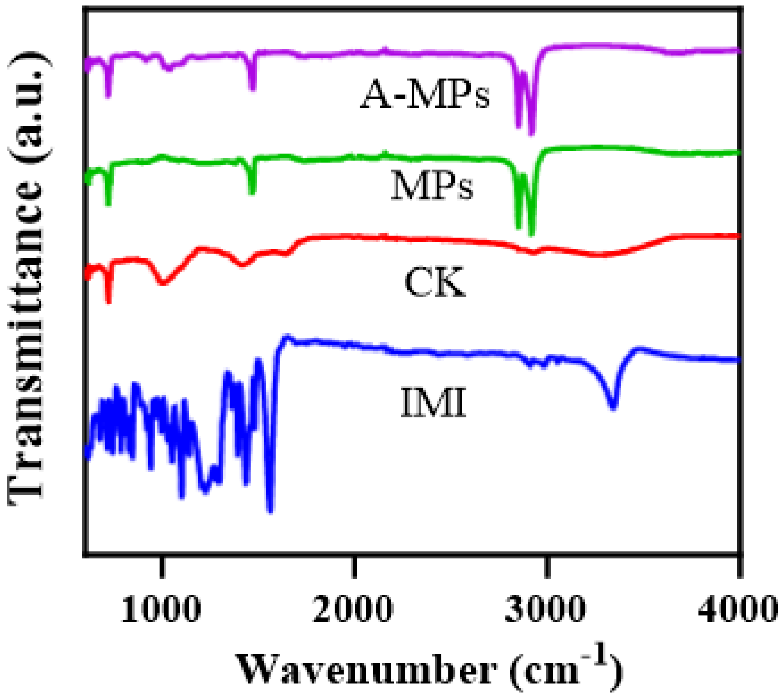

Figure S2. Fourier transform infrared spectroscopy shows aging polyethylene MPs (A-MPs), MPs, CK, IMI.

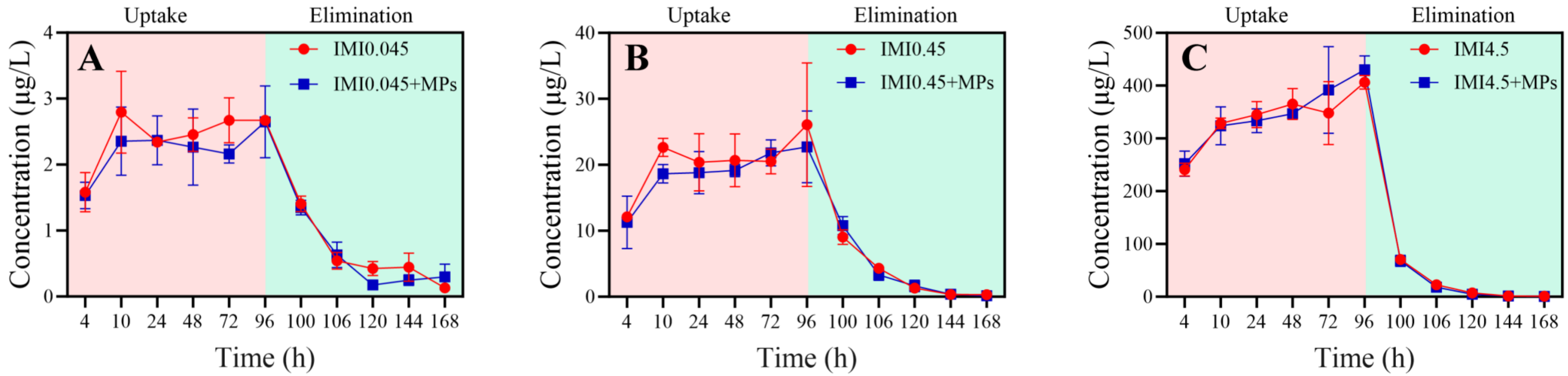

Figure S3. Residual IMI concentrations in tadpoles. (A: low concentration group; B: medium concentration group; C: high concentration group).

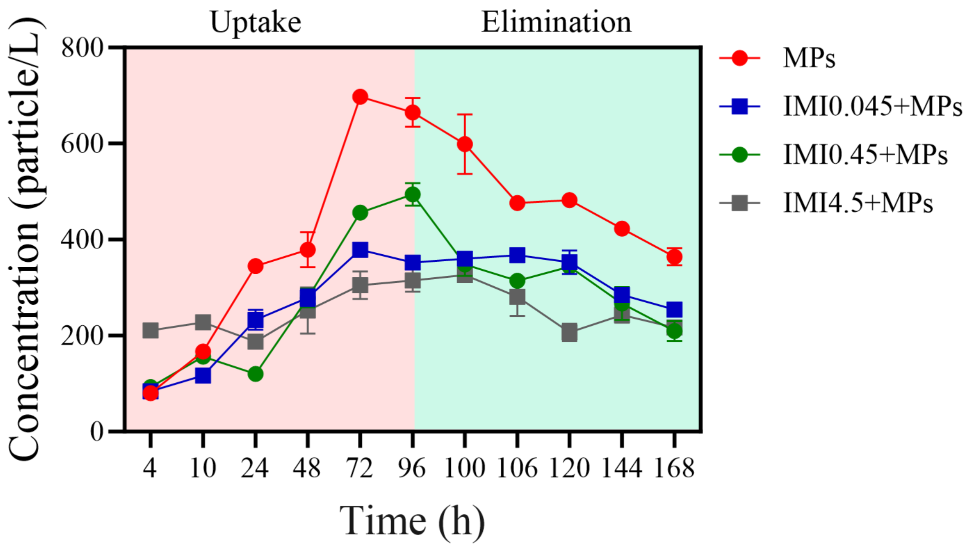

Figure S4. Residual MPs concentrations in tadpoles.

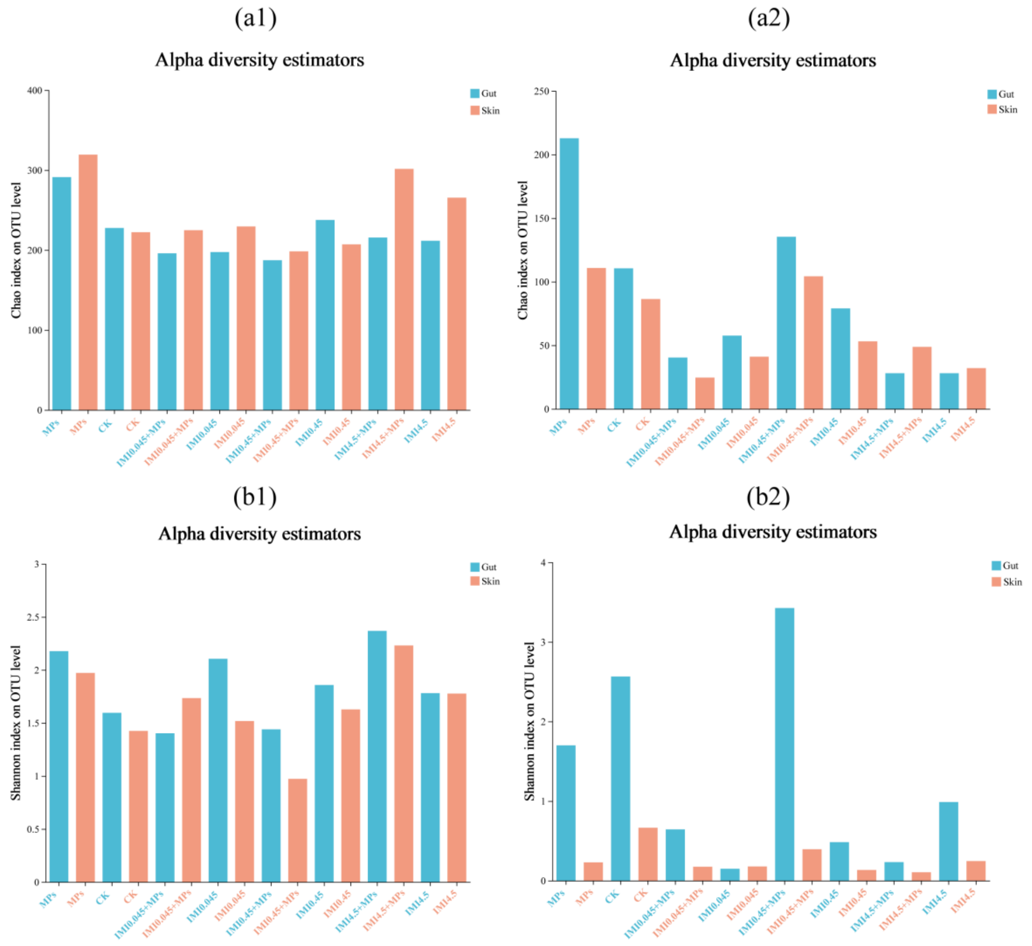

Figure S5. The α diversity in the different exposure groups, including Chao (a) and Shannon indices (b); a1 and b1 represent bacteria, a2 and b2 represent fungi.

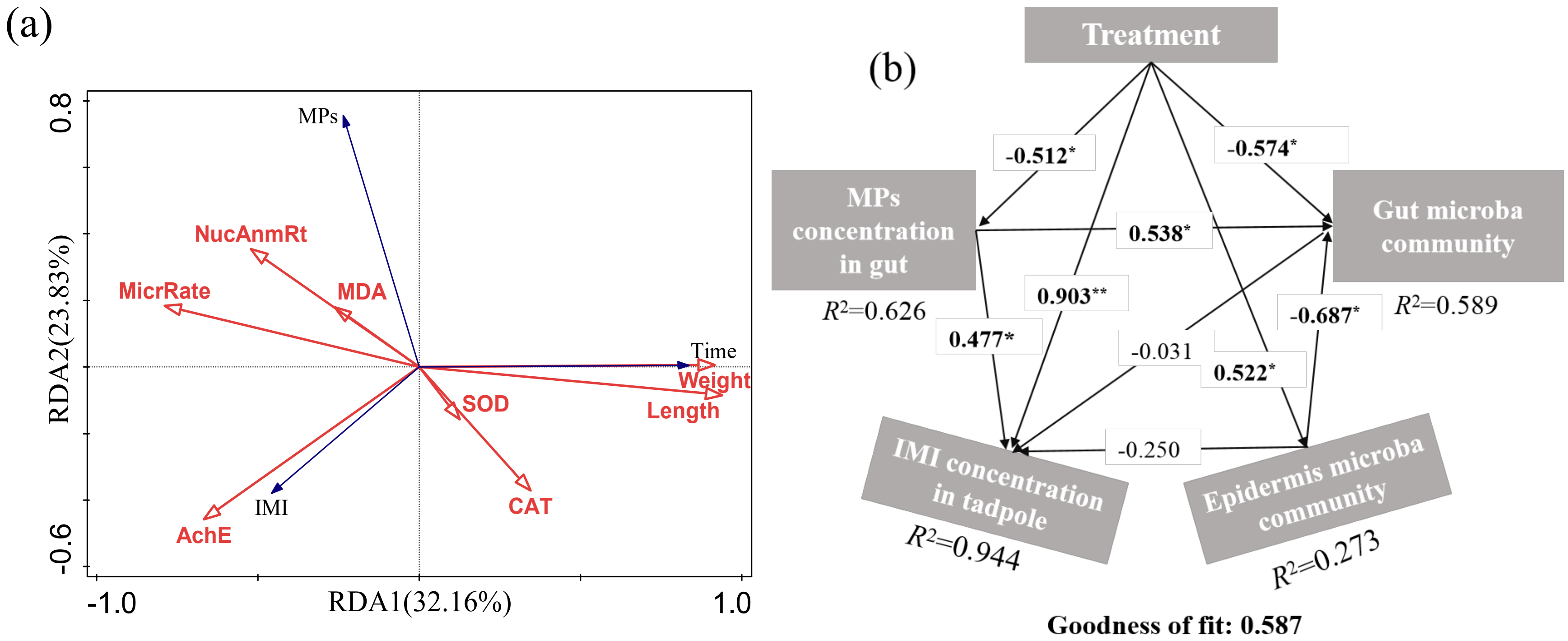

Figure S6. (a) Redundancy analysis (RDA) results for IMI and MPs co-exposure treatment and their toxicity response variables. (b) Numerical values near lines in the structural equation model (SEM) path analysis are standardized path coefficients. Pathway significance: * $p < 0.05$, ** $p < 0.01$, which are indicated by bold font.

Table S1. Physicochemical properties of imidacloprid (IMI).

Name	Abbr.	Chemical structure	CAS	MW[a]	MV [b]	VP [a]	HL constant [a]	S_w [a]	LogK_{ow}[a]	pK_a [a]	K_{oc} [a]
Imidacloprid [a]	IMI	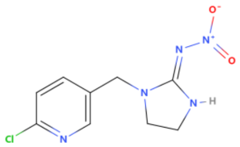	138261-41-3	255.66	204.77	4.0×10^{-07}	1.7×10^{-10}	610	0.57	No dissociation	225

MW: molecular weight (g mol^{-1}); MV: molecular volume ($Å^3$); VP: Vapour pressure at 20°C (mPa); HL: Henry's law constant at 25°C (Pa m^3 mol^{-1}); S_w: solubility in water at 20°C (mg L^{-1}); LogK_{ow}: logarithm of octanol water partition coefficient; pK_a: dissociation constant in water at 25°C; K_{oc}: soil organic carbon normalized apparent partition coefficient (L g^{-1}).

[a] data from PPDB: Pesticide Properties DataBase (https://sitem.herts.ac.uk/aeru/ppdb/en/search.htm), accessed May 31, 2023.

[b] the molecular volume (MV) was predicted by using Interactive property calculator (https://www.molinspiration.com/services/volume.html), accessed May 31, 2023.

Table S2. Mortality of *H. rugulosus* when exposed to the different concentration of IMI in presence or absence of MPs over 24, 48, 72, 96 h.

IMI Concentration ($mg\ L^{-1}$)	Mortality (%)							
	24 h		48 h		72h		96h	
	With MPs	Without MPs	With MPs	Without MPs	With MPs	Without MPs	With MPs	Without MPs
0	0	0	0	0	0	0	0	0
15	4.03	0	4.98	6.25	12.86	12.50	19.41	28.13
45	21.36	3.13	24.42	9.38	42.90	18.75	53.65	46.88
75	36.35	3.13	40.17	15.63	60.44	40.63	70.37	71.88
105	47.73	28.13	51.72	43.75	71.12	46.88	79.60	75.00
135	56.41	43.75	60.32	56.25	78.09	62.50	85.22	84.38
165	63.15	46.88	66.85	59.38	82.88	65.63	88.88	96.88
195	68.47	53.13	71.94	62.50	86.32	81.25	91.39	100.00
225	72.75	62.50	75.97	75.00	88.86	87.50	93.18	100.00

Table S3. Analysis results of the redundancy analysis (RDA) grouped by oxidative stress markers.

**Name**	**Explains %**	**Contribution %**	**pseudo-F**	***P***
Length	28.6	37.3	12.0	0.002
Nuclear anomaly rate	16.4	21.4	8.6	0.002
CAT	8.5	11.1	5.1	0.006
AchE	7.2	9.4	5.0	-
Weight	4.5	5.8	3.3	0.042
Micronucleus rate	4.3	5.6	3.5	-
SOD	3.8	4.9	3.4	0.038
MDA	3.4	4.4	3.3	0.024

pseudo-F: statistical value; ***P*:** Significance of statistical result
